# Forces for Folding

**DOI:** 10.32607/actanaturae.11336

**Published:** 2021

**Authors:** C. Crane-Robinson

**Affiliations:** Biophysics Laboratories, School of Biology, University of Portsmouth, PO1 2DT, UK

**Keywords:** thermodynamics, calorimeters, enthalpy, protein folding, hydration

## Abstract

Understanding the nature of the forces driving the folding of proteins, nucleic acids and the formation of their complexes absolutely requires thermodynamic data, in addition to structural information. In practical terms, this means the use of super-sensitive scanning and titration calorimeters for experimental determination of the heats (enthalpies) characterising these processes. Peter Privalov was both an experimental thermodynamicist and a calorimeter designer/manufacturer who followed and propagated this credo. The sum total of his many publications, every one of which addresses a fundamental question, is his lasting epitaph.

## INTRODUCTION

The first views of the folded globin chains, with a heme group nestling among the many helices, as shown by Perutz and Kendrew in the late ‘50s, was a wonder to behold. Pauling’s α-helix was celebrated beyond expectations. The question as to how a tetramer of such globules fulfilled its functions remained to be elucidated –and Perutz spent the rest of his life doing just that –but the structural underpinnings were very apparent. But not quite to everyone’s full satisfaction, however. Peter Privalov immediately asked himself: “what are the forces that stabilise these helices and drive their folding to generate this complex but exquisite bundle”? Another question was posed by Oleg Ptitsyn: “by what route, i.e. pathway, does the disordered chain convert to the folded globule”? So, new issues were raised, moreover from new directions.

## EXPOSITION

Peter Privalov was the quintessential experimental, scientist who realised that the only way to define the forces responsible was to make thermodynamic measurements, which meant determining the heats of protein folding. At the dilute protein concentrations for which intermolecular effects can be neglected, such heats are extremely small, best arrived at by measuring the heat capacity changes of the solution as the protein undergoes thermal denaturation. So he devised (one could say ‘invented’) a calorimeter of two identical compartments that measured the heat capacity of the protein solution in one, relative to plain buffer in the other as their temperature was raised. The differential scanning calorimeter was thus born [1, 2].

The indicative protein used was lysozyme, and its calorimetric heat of unfolding was measured [3]. The heat input required (by convention a *positive *enthalpy) could be explained by the breaking of H-bonds and van der Waals close contacts, and the rise in the concomitant entropy, a result of the increased conformational freedom of the polypeptide chain. That much seemed clear, but a simpler approach to a determination of the thermodynamic parameters of melting is possible; it is called the van’t Hoff (vH) plot. The sigmoidal melting transition of proteins suggests some degree of cooperativity, and if passage through the transition is plotted against the inverse temperature, this yields the apparent enthalpy and entropy involved: so, why the necessity for a sensitive calorimeter? Unfortunately, the thermodynamic parameters derived from a vH plot indicate nothing about the degree of cooperativity and can be positively misleading. But when the calorimetric enthalpy of lysozyme denaturation was compared with the vH value, they turned out to be actually the same! The correct value from the vH plot meant that the denaturation transition involves *only *two states, which must be the fully folded form and the fully denatured form of the chain. So, lysozyme denaturation is a fully cooperative process in which no other thermodynamically relevant states participate, proving its unfolding to be a genuine “all or nothing” process. The physical description of such a two-state transition is illustrated by the state of the protein in the middle of the unfolding transition: half the sample is still fully folded, and the other half completely unfolded; i.e., it is *not *a homogeneous sample of half-folded macromolecules. Demonstration of protein folding as such a highly cooperative process was a revelatory discovery at that time.

Privalov then turned to larger proteins, such as pepsinogen, and found that the calorimetrically measured melting enthalpy was about twice that obtained from the vH plot [4]. It followed that native pepsinogen, although apparently a unified single fold, in fact consists of two quasi-independent sub-domains, each with its hydrophobic core. Differential scanning calorimetry might have seemed a somewhat obscure methodology in the 1970s and not of prime importance, but Privalov’s observations were direct support for his conviction that solving the 3D structures of proteins was not sufficient for understanding the nature of the forces driving their formation and providing the stability of their native folds: only thermodynamic studies could do that.

Peter Privalov was the quintessential experimental scientist, a thermo-dynamicist extraordinary, who sadly died in Baltimore on December 20^th^ 2020.

His scientific career started in his native Georgia at the Institute of Physics in Tibilisi, doing his Ph.D. under the supervision of its director, the low-temperature physicist Elveter Andronikashvili. His first super-sensitive calorimeter for measuring temperature-induced heat capacity changes in dilute solutions appeared in 1964 [1]. Even at that time, however, he was not quite a lone wolf: Julian Sturtevant was already making calorimetric measurements [5] and was much admired by Privalov, who frequently spoke and wrote very warmly of his influence [6]. In 1967, Privalov was recruited as one of 6 team leaders at the founding of the Institute of Protein Research in Poushchino (a research town 60 miles south of Moscow) – to which Ptitsyn was also recruited. He finally assembled a team to develop a ‘school of bio-thermodynamics’, an essential prerequisite, as he saw it, for taking the subject forward. It also provided the opportunity for instrument development and, before long, the commercial manufacture of a scanning calorimeter (DASM-1). This instrument was unique and consequently sold widely even in Western countries. Export of sophisticated scientific equipment from the USSR at that time was very rare and in stark contrast to the high level of imports such as NMR spectrometers, ultracentrifuges and the like. For this achievement, Privalov won a State Prize in 1978.

The single defining principle of his ‘School’ was to understand the forces controlling the formation, stability, and interactions of biological macromolecules: take a pure sample of the object in question and subject it to a thorough experimental calorimetric analysis. Then, do your best to interpret the results obtained: don’t start with a theoretical analysis, as this might lead to strong convictions as to the expected result and be very prejudicial to performing appropriate experiments. Meaning: theory should follow an experiment, not lead it!

One cause of Privalov’s wariness regarding theoretical descriptions of the driving forces was uncertainty as to how the role of the solvating water was modeled. The significance of hydration was exemplified by noting the large increase in heat capacity (ΔCp) of proteins when they denature and expose the hydrophobic residues of the core to water –the explanation for which was the changed state of the hydrating water molecules. Privalov was always at pains to point out the importance of knowing ΔCp, a quantity that determines the temperature dependence of all the thermodynamic parameters. The role of water very often drove his thinking: in 1979, he published a seminal paper [7] explaining how hydration of the collagen triple helix leads to the temperature dependence of its stability and flexibility.

The dissolution of the Soviet Union as a political entity in 1991 soon led to the collapse of its scientific enterprise and a large-scale exodus of researchers. Privalov was no exception, but unlike the contract posts given to the majority, he was offered a tenured professorship at a top U.S. research university (Johns Hopkins) that he took up in 1992. Despite such a dramatic change of circumstances, he never wavered in his commitment to understanding the basic principles of macromolecular stability: he did not resort to ‘opportunistic science’ and go with the prevailing winds so as to attract funding. Only fundamental questions were asked, and the experiments designed to answer them were then conducted. Nor did he stop developing DSC instrumentation: calorimeters with capillary cells made from gold were constructed. The idea for this metal came to Privalov (he reported) as he sat in the dentist’s chair having a gold crown fitted: what an ideal material, very high thermal conductivity, chemically inert and very malleable. Such instruments were then commercialized in the U.S. Several spectacular scientific achievements from the Hopkins Lab come to mind, such as the ‘cold denaturation’ of proteins [8], accompanied by heat release, and the energetics of folding of an individual α-helix [9]. To these must be added his experimental determination of the entropy associated with forming a dimeric protein from two separate monomers –the so-called translational entropy –that he determined to be much less than predicted; in fact, about one order of magnitude lower than proposed by theoreticians [10, 11].

Privalov’s realization that precision is essential in the measurement of thermodynamic quantities meant that he was not always an easy-going task-master: he expected results of maximum precision and very small tolerance for errors –a challenging requirement. But those results were trustworthy, and the data could be relied upon. Although demanding, Privalov most certainly did not lack a sense of humour. On a visit to a thermodynamics conference organised by the UK Institute of Physics in Salford, the birthplace of James Joule: we bemoaned the fact that whilst our data were always expressed in joules, many participants were still using calories. “Ah yes”, Privalov remarked, “but remember that Joule himself used calories”!

When the 21^st^ century dawned, Privalov was very successfully installed at Hopkins and had switched from individual proteins to DNA and its interactions with the binding domains (DBDs) of transcription factors. Measurements of binding enthalpies were confounded by concomitant refolding of DBDs: Privalov’s important contribution was to demonstrate how a combination of DSC with titration calorimetry can overcome this problem and thereby define the forces giving rise to the fully folded complexes revealed by crystallography [12]. In recent years, the DNA duplex itself became the object of study, joining many other researchers in a popular topic –but a field in the firm grip of the conviction that duplex melting is not accompanied by any change in heat capacity: so, DNA energetics are temperature independent. However, using careful measurements with several short duplexes, Privalov showed this not to be the case, once again the hydrating water playing a critical role [13].

Privalov never let up on his determination to understand the basis of all the forces involved in macromolecular folding and stability, most recently the contribution of hydrogen bonding. Experiments to determine the energetics of formation of the DNA duplex led to the conclusion that the contribution of H-bonds is not enthalpic but comes from the entropy increase resulting from the release of the water molecules bound to the bases in the disordered state of the oligonucleotides [14]. If that is the case, then what can be said about the formation of the H-bonds in α-helices? Is that also an essentially entropic process? In fact, Privalov’s article discussing this last, very fundamental point is currently in press [15].

## CONCLUSIONS

Science is a conversation: its participants tell each other about their results, and the building rises from their combined efforts. Privalov was both diligent and masterful in presenting his results: firstly, in writing many individual articles, followed by several, very extensive reviews of his work on proteins [16, 17], then on protein/DNA interactions [18], and finally in 2012 publishing a whole book “Microcalorimetry of Macromolecules” that splendidly sums up his complete oeuvres [19]. There is so much to be admired in all that Privalov achieved: the sum total of his publications is his epitaph. We have lost a great experimentalist of the highest stature, devoted to basic science, and we are all much the poorer for it.
